# Beneficial and Paradoxical Roles of Anti-Oxidative Nutritional Support for Non-Alcoholic Fatty Liver Disease

**DOI:** 10.3390/nu10080977

**Published:** 2018-07-27

**Authors:** Daisuke Uchida, Akinobu Takaki, Takuya Adachi, Hiroyuki Okada

**Affiliations:** Department of Gastroenterology and Hepatology, Okayama University Graduate School of Medicine, Dentistry and Pharmaceutical Sciences, 2-5-1 Shikata-cho, Kita-ku, Okayama 700-8558, Japan; d.uchida0309@gmail.com (D.U.); adataku719@yahoo.co.jp (T.A.); hiro@md.okayama-u.ac.jp (H.O.)

**Keywords:** non-alcoholic fatty liver disease, hepatocellular carcinoma, anti-oxidant

## Abstract

Oxidative stress is being recognized as a key factor in the progression of chronic liver disease (CLD), especially non-alcoholic fatty liver disease (NAFLD). Many NAFLD treatment guidelines recommend the use of antioxidants, especially vitamin E. Many prospective studies have described the beneficial effects of such agents for the clinical course of NAFLD. However, as these studies are usually short-term evaluations, lasting only a few years, whether or not antioxidants continue to exert favorable long-term effects, including in cases of concomitant hepatocellular carcinoma, remains unclear. Antioxidants are generally believed to be beneficial for human health and are often commercially available as health-food products. Patients with lifestyle-related diseases often use such products to try to be healthier without practicing lifestyle intervention. However, under some experimental NAFLD conditions, antioxidants have been shown to encourage the progression of hepatocellular carcinoma, as oxidative stress is toxic for cancer cells, just as for normal cells. In this review, we will highlight the paradoxical effects of antioxidants against NAFLD and related hepatocellular carcinoma.

## 1. Introduction

Non-alcoholic fatty liver disease (NAFLD) is a common chronic liver disease associated with obesity and metabolic syndrome [1,2]. The deposition of lipids in the liver induces inflammation, insulin resistance, and hepatic steatosis [3]. Non-alcoholic steatohepatitis (NASH) is a severe form of NAFLD that causes cirrhosis and hepatocellular carcinoma (HCC) because of persistent inflammation associated with hepatic steatosis [4,5]. The mechanism underlying the development of NASH from simple steatosis, namely non-alcoholic fatty liver (NAFL), has been considered as either a two-hit theory or a multiple hit-process [6,7,8]. The two-hit theory comprises a first hit of hepatic steatosis and second hit of several cellular stress responses, such as oxidative stress or endoplasmic reticulum (ER) stress. Given that the second hits are multifactorial and hepatic steatosis may be induced via hepatic inflammation, the process has recently been expressed as a multiple parallel hit process or multiple hit process [8]. 

Oxidative stress is a cellular stress associated with NASH progression. Reactive oxygen species (ROS) are generated during free fatty acid metabolism in microsomes, peroxisomes, and mitochondria, and comprise an established source of oxidative stress [9]. Oxidative stress is increased through the generation of ROS, as well as by defects in redox defense mechanisms involving glutathione (GSH), catalase or superoxide dismutase (SOD), and it induces various events related to not only NASH progression but also carcinogenesis such as DNA damage, tissue remodeling, and alterations in gene expression [10,11]. Mitochondria play a key role in the development of oxidative stress, and the dysfunction of mitochondria may induce NASH progression. Some reports have shown that antioxidant therapies, such as vitamin E, improve NASH, but the mechanism, especially concerning malignant tumor progression, and long-term outcomes are not clear [12,13]. 

In the present study, we reviewed the relationship between antioxidant nutrients and NASH progression including the carcinogenic risk. 

## 2. The Relationship between NAFLD and Oxidative Stress 

Oxidative stress is strongly related to chronic hepatic inflammation, and NAFLD/NASH is no exception, being included in the “second hit” of NASH progression as well as apoptosis, ER stress, and intestinal environment. It is widely known that oxidative stress functions as an important regulator of the progression of liver steatosis [14,15,16]. Various cellular stresses, including oxidative stress, apoptosis, and gut-derived signals such as lipopolysaccharide (LPS), trigger an inflammatory response and progressive liver damage [17]. Chronic oxidative stress correlates with a variety of pathologies such as malignant diseases, diabetes mellitus, cardiovascular diseases, chronic inflammatory diseases, and aging acceleration. Oxidative stress is caused by the generation of ROS, which have various causes, including interactions among gut microbiota. When excess ROS are produced excessively or the endogenous antioxidant capacity is diminished, indiscriminate oxidation elicits harmful effects resulting in oxidative stress [18]. 

Insulin resistance and dyslipidemia are widely accepted as disease progression factors in NAFLD [7]. Insulin signals suppress gluconeogenesis and enhance lipid synthesis in the liver. A human hepatic mRNA analysis revealed that the expression of insulin receptor substrate (IRS)-2, which mediates the effects of insulin by acting as a molecular adopter, was decreased while that of gluconeogenesis enzymes was increased in both NAFL and NASH [19]. Insulin resistance is clinically evident in the advanced stage of NAFLD; however, hepatic insulin resistance at the molecular level started from the NAFL stage. Altered cholesterol homeostasis such as increased cholesterol synthesis and uptake or reduced cholesterol excretion have been shown in NAFLD [20]. The hepatic free cholesterol accumulation has been shown to damage mitochondria and ER function thereby inducing hepatic inflammation and fibrosis [21]. Lipoprotein lipase (LPL), a key regulator of fatty acid release from triglyceride-rich lipoproteins, controls the cellular uptake of fatty acid and triglyceride accumulation [22]. The inhibition of LPL may prevent the accumulation of hepatic lipid [23].

Changes in the alimentary tract environment, which is connected to the liver via the portal vein, including the oral microbiota to the intestinal and colorectal microbiota have been shown to be strongly related to various diseases. The gut microbiota, which comprise various species such as *Bifidobacterium*, *Bacteroides*, *Clostridium*, *Lactobacillales,* and *Prevotella*, produce endogenous ethanol that induces the formation of ROS by hepatic stellate cells and Kupffer cells [24]. The composition of the microbiota depends on the age, health history, food intake, and probiotics consumed [25]. The food intake strongly correlates with dyslipidemia and is a crucial factor to consider when deciding on treatments for NAFLD, as it influences the gut microbiota [26]. 

An imbalance in the microbiome can affect the intestine and liver via microbiota-related signaling pathways such as toll-like receptor (TLR) signaling activation. LPL can also be suppressed by a fasting-induced adipose factor, which is regulated by the gut microbiota via TLR ligands [7,27]. A TLR-4 ligand LPS is liberated from the outer membrane of Gram-negative bacteria and binds to lipopolysaccharide binding protein (LBP). These ligands stimulate TLR signals, including nuclear factor kappa (NF-kB) with cluster of differentiation (CD) 14, and induce the production of inflammatory cytokines [28]. LPS is increased by a high-fat, high-calorie diet, as well as dyslipidemia and insulin resistance, which are related to NASH progression. The involvement of TLR-2 in NASH inflammation and fibrosis has been investigated in mouse NASH models [29]. A serum multiple cytokine analysis revealed that the levels of the chemokine interferon gamma inducible protein 10 (Interferon gamma-induced protein (IP)-10; C-X-C motif chemokine (CXCL)-10 increased stepwise from healthy volunteers to NAFL and NASH patients. A TLR-2 ligand peptidoglycan in combination with an in vitro insulin resistance condition (i.e., high glucose with insulin in the culture medium) increased the expression of IP-10 mRNA, indicating the importance of insulin resistance with bacterial signals in NASH progression [30]. Therefore, the diet helps to establish a close relationship among the gut microbiota, oxidative stress and NAFLD/NASH. 

## 3. Treatments for NAFLD/NASH 

The treatment strategy of NAFLD involves intervention with diet, exercise, and drugs [13,31,32,33,34]. Lifestyle interventions, including diet and exercise, are strongly related to insulin resistance and dyslipidemia. A randomized, controlled trial testing simple weight loss gain by lifestyle intervention using a combination of diet, exercise, and behavior modification resulted in NASH pathological improvement [35]. Insulin resistance has been shown to be an independent predictor of NASH in biopsy-proven cases that were likely to be controllable by any method, including lifestyle intervention [36]. Dyslipidemia has various causes, such as hereditary predisposition, a sedentary lifestyle, and a high calorie intake. The improvement of dyslipidemia may resolve NASH progression, and nutritional support, such as low-fat and low-calorie diets, holds a prominent position in treatment approaches [37,38]. 

Vitamin E is the most commonly used antioxidant treatment for NASH [39]. Pioglitazone, an antihyperglycemic drug, is reported to ameliorate liver injury and fibrosis in patients with NASH with and without diabetes [33]. Sanyal et al. reported that vitamin E therapy was effective for NAFLD improvement compared with pioglitazone and placebo and recommended antioxidant treatment as well as lifestyle intervention, bariatric surgery, dyslipidemia treatment, and diabetes treatment [13]. On the basis of these data, the American Association for the Study of Liver Diseases (AASLD) recommends treating NASH with vitamin E [40]. However, the Treatment of NAFLD in Children (TONIC) randomized trial showed that neither metformin nor vitamin E improved the liver function in patients with NAFLD [41]. A meta-analysis of randomized controlled trials revealed that vitamin E and pioglitazone but not metformin improved the histological activity of NASH [42]. However, the efficacy of these drugs is still controversial, and further clinical studies including assessments of long-term outcomes should be conducted.

Probiotics are reported to be a promising treatment option for NAFLD, improving the liver function, obesity, and insulin resistance [43]. Shavakhi et al. reported that metformin in combination with probiotics improved liver inflammation to a significantly greater degree than metformin alone [44]. This effect may be caused by the association between the gut microbiota and liver, which receives blood from the intestine through the portal vein. 

Surgical treatments, such as bariatric surgery and liver transplantation, are conceivable treatments for NAFLD/NASH. Bariatric surgery for obesity is effective for improving NASH, as weight loss can improve dyslipidemia and hepatic fibrosis [45,46]. It also decreases the mortality rate due to diabetes, cardiology events, and malignant tumors. Liver transplantation is another treatment option for NAFLD/NASH related end-stage cirrhosis [47]. However, NASH can occur after liver transplantations, and continuous medical approaches are needed [48].

## 4. Carcinogenesis and Cancer Progression in Correlation with Oxidative Stress and Antioxidant Treatment

Hepatocellular carcinoma (HCC) is the major malignant liver tumor and the main cause of death in NAFLD/NASH patients. Oxidative stress is strongly associated with hepatocarcinogenesis and cancer progression. However, the effects on cancer-free patients and patients with HCC are likely different. Oxidative stress has a driving effect on carcinogenesis while also exerting toxicity against cancer cells. This contradiction will be discussed in this section. 

### 4.1. Oxidative Stress in NASH-Hepatocarcinogenesis

Oxidative stress is reported to induce various events related to carcinogenesis such as DNA damage, tissue remodeling, and alterations in gene expression [11,49,50]. ROS production is stimulated by inflammatory factors, and oxidative stress promotes oncogenic transformation due to DNA-damage. ROS activates various pathways, such as Wingless-type MMTV intefration site family (Wnt) /β-catenin, NF-kB, or myc proto-oncogene protein (c-Myc)/Transforming growth factor (TGF)-α. This activation also occurs in carcinogenesis in liver cancer. Inducible nitric oxide synthase (iNOS), which produces nitrogen monoxides (NO), correlates with chronic inflammation, cell proliferation, DNA repair, migration, and angiogenesis [51]. The iNOS activity is important for endothelial stress gene expression, which protects against oxidative stress, and may be the key modulator of hepatocarcinogenesis. 

8-hydroxydeoxyguanosine (8-OHdG), which is produced by DNA-damage with ROS, reflects the amount of oxidative stress and generates a point mutation in DNA double strands. Kawanishi et al. reported that 8-OHdG, which is a reliable marker for oxidative stress in the liver, strongly correlates with infection-related carcinogenesis via chronic inflammation [52]. Seki et al. reported that the 8-OHdG expression in the liver tissue is related to the pathological features of NAFLD and hepatocarcinogenesis [53]. 

Adenosine monophosphate activated protein kinase (AMPK) is a highly conserved heterodimeric serine-threonine kinase that plays a role in cellular energy homeostasis and different fundamental cellular processes such as the cellular proliferation, survival, and metabolism. It serves as an energy sensor in eukaryotic cells and bridges metabolism to carcinogenesis [54]. Its activated form (phospho (p)-AMPK) is down-regulated in HCC tissues from patients and low levels of p-AMPK correlate with a poor prognosis, indicating the importance of AMPK signaling in HCC [55]. AMPK signaling has recently been indicated to correlate with inflammatory responses, as the energy metabolism in immune cells is related to immunoregulation [56]. Under many oxidative stress-related conditions, such as diabetes or dyslipidemia, a diminished AMPK activity has been shown to be associated with tissue oxidative stress [57,58]. 

As mentioned above, oxidative stress is linked to the progression from NAFLD to HCC. The correlation of AMPK and clinical conditions encourage us to control AMPK as a cancer regulator.

### 4.2. Antioxidant Treatment Effects on Prevention of NASH-Hepatocarcinogenesis 

Given that advanced chronic liver disease is the strongest risk factor for hepatocarcinogenesis, antioxidant treatments can be an important approach to preventing HCC development from NASH. The effect of these agents concerning whether they have the properties to protect against hepatocarcinogenesis should include an improvement in liver fibrosis improvement.

Vitamin E was reported to prevent hepatocarcinogenesis via the down-regulation of iNOS and nicotinamide adenine dinucleotide phosphate (NADPH) oxidase [59]. Many clinical studies have reported that vitamin E improves NAFLD and prevents the development of HCC [13,60,61,62]. However, these studies were all performed over a relatively short period (i.e., within two years). Hepatocarcinogenesis progresses from NAFL/NASH to cirrhosis over a long period of time, and middle-aged men carry risks of hepatocarcinogenesis even at an early stage of NAFLD [63]. The long-term outcomes must be evaluated in order to judge the true efficacy of antioxidant therapy for NASH-hepatocarcinogenesis. 

Many candidate antioxidant agents may show favorable effects on NASH and NASH-related hepatocarcinogenesis.

Anti-diabetic agents such as metformin, pioglitazone, glucagon-like peptide 1-receptor agonists (GLP-1 RAs), and dipeptidyl-peptidase-4 inhibitors (DPP-4Is) were recently found to show favorable effects on the NAFLD clinical course [7,64,65]. Metformin and pioglitazone in particular are regarded as anti-oxidant agents. Metformin is the most well-studied AMPK-activating agent. Metformin-related AMPK pathway activation is involved in many cell types including T cells, B cells, hepatocytes, and even liver fibrosis-inducing hepatic stellate cells (HSCs). In in vitro and in vivo models (mice), metformin suppressed alpha smooth muscle actin (α-SMA) expression via AMPK activation and the inhibition of the succinate-related pathway in (HSCs) [66]. HSC activation is an important step in hepatocarcinogenesis. In addition, metformin induces the production of the antioxidant enzyme, heme oxygenase-1 (HO-1) in human endothelial cells via the nuclear factor erythroid 2-related factor (Nrf2) signaling pathway [67]. Determining metformin’s long-term effects on reducing the risk of hepatocarcinogenesis might require further long-term studies.

Pioglitazone is a thiazolidinedione activating peroxisome proliferator-activated receptor (PPAR) γ that improves the insulin resistance and is used to treat type 2 diabetes. A six-month randomized study of pioglitazone revealed a reduction in necroinflammation of liver histopathology with no reduction in fibrosis [68]. l-carnitine is an essential nutrient and an important molecule in regulating mitochondrial and peroxisomal metabolism [69]. A recent randomized controlled study indicated histological improvement of liver fibrosis after 18 months with pioglitazone for NASH in patients with concomitant type 2 diabetes [70]. This agent may therefore be effective for inhibiting NAFL progression to NASH and hepatocarcinogenesis. 

Flavonoids are heterogeneous polyphenols found in various plants, such as fruits, vegetables, and green tea that reportedly exert antioxidative function protecting the liver tissue from damage caused by ROS [71]. A mixture of flavonolignans and minor polyphenolic compounds, derived from the milk thistle plant (*Silybum marianum*) known as silymarin, is said to be an antioxidative agent [72]. Salomone et al. reported that silibinin, the main component of silymarin, restored nicotinamide adenine dinucleotide (NAD+) levels, and played a protective role against NAFLD [73]. In a randomized, double-blind, placebo-controlled study, silymarin at 700 mg three times daily for 48 weeks significantly ameliorated liver fibrosis in NASH patients [74]. Silymarin may therefore be a promising agent for the treatment of NAFLD/NASH patients.

l-carnitine acts as an antioxidant mediator by controlling the β-oxidation cycle and adenosine triphosphate (ATP) generation [75]. l-carnitine is well known as a fat-burning supplement; however, it has recently attracted attention as an anti-cancer agent because of its effects as an antioxidant, apoptosis inducer, and inhibitor of histone deacetylase [76,77,78,79]. We previously reported that l-carnitine controls the mitochondrial function in hepatic non-cancerous tissue from a mouse NASH model by upregulating the *Lactobacillales* population related to the secondary bile acid production in the gut microbiota [76]. l-carnitine does not eliminate oxidative stress but rather controls the oxidative balance to aid the mitochondrial function. This might be a better approach to regulating oxidative stress.

High levels of plasma free fatty acids increase the levels of hepatic free fatty acids. Long-chain fatty acids taken up by mitochondria as complexes with l-carnitine are subsequently metabolized in β-oxidation pathways. Under oxidative stress, oxidative reactions convert oxidized cofactors (NAD+ and flavin adenine dinucleotide (FAD)) into reduced cofactors (nicotinamide adenine dinucleotide (NADH) and flavin adenine dinucleotide H2 (FADH2)) and deliver electrons to the respiratory chain. An imbalance between the increased delivery of electrons to the respiratory chain and the decreased outflow from the respiratory chain causes electrons and ROS products to accumulate. Antioxidant defenses, such as superoxide dismutase (SOD), glutathione peroxidase (GPx), or catalase, can metabolize O_2_· and H_2_O_2_ to non-toxic H_2_O. However, the Fenton and/or Haber-Weiss reactions generate highly reactive, toxic, hydroxyl radicals (OH). Vitamin E as a general cytotoxic ROS scavenger erases oxidative stress. Metformin activates AMPK and induces antioxidant gene transcription, and 5-aminoimidazole-4-carboxamide-1-β-d-ribofuranoside (AICAR) activates Nrf2 via a mechanism possibly similar to that of metformin. l-carnitine supports the mitochondrial function to increase the long-chain fatty acid uptake. In NASH patients, oxidative stress works as toxic from NASH progression to cancer development. However, in cancer patients, oxidative stress should be maintained to some degree in order to control cancer via toxic oxidative stress reactions. To control cancer progression, AMPK/mammalian target of rapamycin (mTOR) signaling has an important function that would be a target to intervene.

### 4.3. Antioxidant Treatment Effects on Liver Cancer 

Various treatments have been proposed for HCC, such as surgical approaches (e.g., liver resection, liver transplantation), radio frequency ablation, transcatheter arterial chemoembolization, and chemotherapy [80]. However, HCC remains incurable because of the low response rate and frequent recurrence based on chronic liver inflammation and fibrosis. Many reports have described the usage of antioxidant therapies for HCC, but their efficacy still remains unclear [81,82,83]. 

Metformin inhibits the cell growth of HCC by regulating AMPK activation and inducing apoptosis [84]. The effect of metformin on NASH-related HCC in mouse models has been reported, but the clinical effect in humans is unclear [85]. 

The AMPK activator 5-aminoimidazole-4-carboxamide-1-β-d-ribofuranoside (AICAR) activates Nrf2 protein, which binds to Kelch-like ECH associated protein 1 (Keap1), a protein that exists in the cytoplasm in an inactive form [86]. AICAR may be a candidate for treating liver cancer. 

Flavonoid and polyphenols have been shown to exert antitumor effects through the control of various molecular pathways [81,87]. Curcumin is an antioxidant polyphenol compound reported to exert antitumor activities by inhibiting the vascular endothelial growth factor (VEGF) expression and phosphoinositide 3-kinase (PI3K)/v-Akt murine thymoma viral oncogene (Akt) signaling [82]. The newly confirmed antioxidant silymarin has also been analyzed for its utility in preventing hepaticarcinogenesis [73].

l-carnitine is a mitochondria supporting agent that may be useful for controlling oxidative stress in HCC patients. Our previous report showed that the tumor number and maximum tumor size were decreased in NASH-HCC mouse models than control diet [76]. In that experiment, l-carnitine was administered from the early onset of NASH, and the liver tumor outcomes were assessed afterwards. Under such conditions, l-carnitine improved the NASH pathogenesis resulting in decreased NASH related hepatocarcinogenesis and so is difficult to conclude whether its use is beneficial for HCC. To answer this point, we next examined the effect of l-carnitine administration after hepatocarcinogenesis in NASH-related cholangiocarcinoma-like tumors [88]. l-carnitine resulted in a good outcome for this model, suggesting it might be an effective antioxidant even after cancer development.

**Figure 1:**
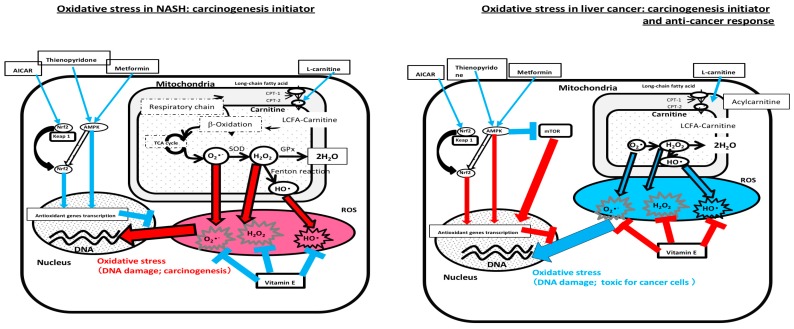
Oxidative stress and anti-oxidant in non-alcoholic steatohepatitis (NASH) and liver cancer. Red lines; unfavorable effects, Blue lines; favorable effects, AICAR, 5-aminoimidazole-4-carboxamide-1-β-d-ribofuranoside; Nrf2, nuclear factor erythroid 2-related factor; AMPK, AMP-activated protein kinase; Keap1, Kelch-like enoyl-CoA hydratase (ECH) associated protein; Nrf2, nuclear factor erythroid 2-related factor; CPT, carnitine palmitoyltransferase; SOD, superoxide dismutase; GPx, glutathione peroxidase; LCFA-carnitine, long-chain acylcarnitine; TCA-cycle, tricarboxylic acid cycle.

As above, many candidate antioxidant treatments have been proposed for HCC; however, the clinical investigations on this point are inadequate at present. To administer antioxidants for cancer stages, we must consider the oxidative stress-related environment adequately (Figure 1).

Recently, oral chemotherapeutic multi-kinase inhibitors such as sorafenib have been widely used to control advanced-stage HCC. Sorafenib inhibits tumor cell proliferation and angiogenesis through the inactivation of vascular endothelial growth factor receptor (VEGFR), platelet-derived growth factor receptor (PDGFR), and the serine-threonine kinase Raf-1, which participates in the Rat sarcoma (Ras)/rapidly accelerated fibrosarcoma (Raf)/mitogen-activated protein kinase kinase (MEK)/mitogen-activated protein kinase (MAPK) signal cascade [89]. Recently, other multi-kinase inhibitors such as lenvatinib, regorafenib, cabozantinib, and ramucirumab have also been developed [90,91,92]. Recent clinical experience with sorafenib has indicated that acquired drug resistance can occur over a long period of drug administration. Thus, in these stages of HCC, the effects of accompanying agents such as antioxidants on the drug resistance should be investigated. Accumulating data indicate that autophagy and phosphoinositide 3-kinase (PI3K)/Akt signaling are associated with acquired resistance to sorafenib in HCC. The AMPK/mTOR signaling pathway had been demonstrated to be important in autophagy. A high sustained glucose condition produces advanced glycation products (AGEs) which damage many organs including the liver. The receptor for AGE (RAGE) has been shown to be overexpressed in HCC promoting proliferation. In addition, RAGE induced sorafenib resistance in in vitro and in vivo xenograft models [93]. A reduction in RAGE was also found to be able to increase autophagy and eliminate sorafenib resistance in vitro via the AMPK pathway activation. Metformin was able to reduce RAGE expression and helped rescue cells from sorafenib resistance. Metformin might therefore be useful for eliminating sorafenib resistance in HCC; however, one clinical study reported a bad outcome for patients administered metformin with sorafenib [94].

Administering sorafenib efficacy-supporting agents is an approach to resolving drug resistance as mentioned above with metformin. The oxidative stress-inducing agent tetrandrine, a bisbenzylisoquinoline alkaloid, has been shown to exert synergistic anti-tumor activity with sorafenib by increasing ROS [95]. More data on such combination therapy with sorafenib will be required to draw more solid conclusions.

Immune checkpoint molecules including programmed cell death-1 (PD-1) and programmed cell death-1 ligand (PD-L1), are promising new therapeutic agents for the treatment of various cancers including HCC [96]. Nivolumab, an anti-PD-1 antibody, was approved by the Food and Drug Administration (FDA) and is a key drug for the treatment of HCC dramatically improving the survival of many cancers, including solid-tumors; however, 30 to 50% of patients are unresponsive [97]. To overcome this unresponsiveness, several combination treatments have been attempted. An in vivo analysis of CD8+ T cells treated with PD-1 antibody revealed that a highly proliferative fraction could be found in the draining lymph node indicating that tumor priming was necessary for CD8+ T cell activation [98]. These highly proliferative CD8+ T cells contained more cellular ROS, a larger mitochondrial mass, higher mitochondrial potential, and more mitochondrial superoxide than CD8+ T cells without PD-1 antibody treatment, indicating the activation of mitochondria in T cells. Several mitochondrial function activators have been tested for their additive effects of PD-1 blockade with successful results. Of them, the PPAR-α agonist bezafibrate showed a particularly promising response. Given that bezafibrate is a standard agent for treating high triglyceride levels, this combination might be useful for combination therapy. The relationship between oxidative stress, antioxidants, and PD-1/PD-L1 needs further study and clinical trials to resolve the issue of a lack of responsiveness to anti-PD-1 antibody therapy.

### 4.4. Cautions of Antioxidants on Liver Cancer Initiation and Maintenance

Whether or not antioxidant agents prevent cancer risks remains controversial. The U.S. Preventive Services Task Force (USPSTF) stated that the current evidence is insufficient to assess the balance of benefits and harms of vitamins [99]. In particular, excessive doses of vitamins may induce negative effects. Four randomized controlled trials (RCTs) of vitamin E included in the USPSTF comments showed no significant effect on the incidence of all types of cancer or cancer mortality rates. However, one RCT assessing the risk of prostate cancer with vitamin E administration found a 17% increase in the prostate cancer incidence with this supplement [100]. Since a large prospective study to confirm the HCC risks for NASH is difficult to perform and requires a long observation time (although there are some ongoing studies), we should attempt to determine the optimum treatment approach based on short-term data from small populations and experimental data.

Oxidative stress is a crucial event for cancer initiation; however, it also has important roles in cancer prevention. Stem cell-like cancer cells have powerful antioxidative properties that protect them from oxidative stress and thus prevent self-apoptosis [101]. The expression of CD44 variant 9 (CD44v9) has recently been investigated as a functional marker of stem-like cells in many types of cancers [102]. The interaction of CD44v9 with xc- Substrate-specific Subunit (xCT), also called the glutamate/cysteine antiporter solute carrier family 7 member 11 (SLC7A11), a subunit of the glutamate-cystine transporter system xc-, stabilizes the latter and thereby potentiates cancer cells to promote glutathione synthesis and reduce the activity of cellular redox system, resulting in resistance to toxic oxidative stresses [103]. Inducing oxidative stress in cancer patients is an approach that is being investigated as a cancer treatment in several clinical trials [104]. However, this approach is likely to also be toxic to normal cells and may lead to the induction of further carcinogenesis. Furthermore, given that treatment-resistant cancer stem cells can still escape toxic oxidative stress, maintaining moderate oxidative stress is a critical point of anti-oxidative stress treatment for cancer patients.

Mitochondrial function-supporting agents, such as metformin or l-carnitine, might be good therapies to investigate in large-scale prospective studies in humans. In cancer patients, the drug effects should be analyzed according to the stages of the cancer. When tumors have been completely resected or ablated, a standard anti-oxidant approach may be effective for preventing new hepatocarcinogenesis. When tumors have not been ablated completely but can be managed with local treatment, such as transcatheter arterial chemo-embolization (TACE), mitochondrial function-supporting agents, such as metformin or l-carnitine, might be useful approaches for controlling the oxidative-antioxidative stress balance. When tumors are not being treated with local therapies but instead managed with anti-cancer agents, presently multi-kinase inhibitors such as sorafenib, regorafenib and lenvatinib, oxidative stress may be an important cellular stress to cancer cells not to be eliminated. As mentioned in previous section, oxidative stress-inducing agents may compound the effects of sorafenib [95]. However, even the favorably reported antioxidant metformin might adversely affect the clinical outcome in patients with HCC receiving the multi-kinase inhibitor sorafenib [94].

l-carnitine is a candidate drug that has shown good efficacy even after cancer has already developed. We previously investigated the effects of the standard anti-oxidant agents vitamin E and l-carnitine and found that only l-carnitine reduced the number of liver tumors [88]. Vitamin E administration induced HO-1 protein expression in cancer tissue, resulting in an increase in the number of stem-like cancer cells. Furthermore, l-carnitine administration improved the *Lactobacillales* population and the balance of bile acid (primary to secondary bile acid), while vitamin E did not affect the intestinal environment. These findings show that vitamin E strongly ameliorates the oxidative stress in both cancer cells and normal cells, while l-carnitine works as an “oxidative stress balancer”. An “oxidative stress eraser” such as vitamin E might therefore be unsuitable for the treatment of cancer patients. However, even l-carnitine carries the possibility of an increased risk of experimental HCC [105]. A mouse HCC model of diethylnitrosamine (DEN)-injected mice fed a high-fat diet showed the predominance of acylcarnitine species in the tumors according to a metabolomic profiling analysis. Intracellular long-chain fatty acids are esterified to acyl-coenzyme A and conjugated with carnitine resulting in acylcarnitine by carnitine palmitoyltransferase 1 (CPT 1). The acylcarnitine is then converted back to acyl-coenzyme A by CPT2 and enters the β-oxidation pathway. Of note, the CPT2 expression was diminished in the HCC tumors inducing acylcarnitine deposition. In this mouse model, l-carnitine supplementation resulted in an increased amount of acylcarnitines and enhanced HCC tumorigenesis. These conflicting results suggest that the HCC-preventive effects of l-carnitine might require a preserved CPT2 function in order to avoid inducing the accumulation of acylcarnitines.

**Figure 2:**
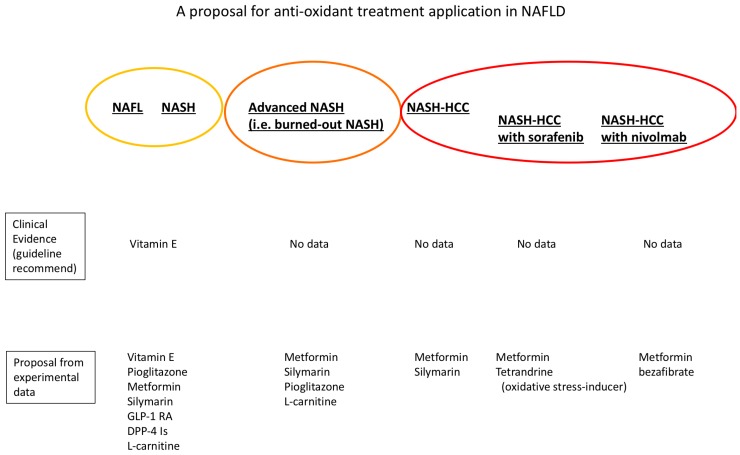
A proposal for the antioxidant treatment application in non-alcoholic fatty liver disease (NAFLD). NAFL, non-alcoholic fatty liver; NASH, Non-alcoholic steatohepatitis; HCC, hepatocellular carcinoma; GLP-1 RA, glucagon-like peptide 1-receptor agonist; DPP-4 Is, dipeptidyl-peptidase-4 inhibitors.

It should be noted that almost all antioxidants can elicit unfavorable effects on carcinogenesis under certain conditions. We must bear these facts in mind and thus perform antioxidant supplementation judiciously. We can use antioxidant for NASH, while should decide whether to stop after HCC development (Figure 2).

Several guidelines recommend vitamin E be administered for NASH, as some prospective studies proved to be beneficial for laboratory data and histological activity. However, there are no prospective data on NASH with diminished activity and steatosis with advanced fibrosis, such as burned-out NASH. There are also no data on NASH-HCC. Many experimental studies recommend antioxidant agents be administered for patients with NAFL and NASH. Given that advanced NASH often shows no steatosis, some antioxidants may be more suitable for these patients than others. For NASH-HCC, given that oxidative stress is an important stress response for regulating cancer cells, strong antioxidants may actually be harmful. When we administer oxidative stress-related agents together with sorafenib and other multi-kinase inhibitors, combination effects may be exerted by oxidative stress-inducing agents. The AMPK activator metformin has been shown to be beneficial for HCC, although clinical evidence is still being discussed. Nivolmab is a next-step HCC treatment approach for activating the cancer immune response. Bezafibrate has been shown to exert additive effects to activate the T cell response.

## 5. Conclusions

NAFLD is a major lifestyle-related disease, and nutritional support is an important approach to its treatment. Antioxidant therapy, such as vitamin E administration, is effective for preventing NASH progression; however, the long-term outcomes of this therapy are unclear, especially in precancerous and cancer patients. Antioxidant therapy should be prescribed according to the clinical condition of the patients, such as those with NAFL, NASH, advanced NASH, cancer with successful ablation, cancer with relative ablation, and advanced cancer requiring anti-cancer agents. In HCC patients, antioxidant treatment should be carefully managed, as these agents can also act against tumor resolution.
